# Unusual cervicofacial localization of two subcutaneous tuberculous cold abscesses in an immunocompetent subject

**DOI:** 10.1016/j.amsu.2021.102551

**Published:** 2021-07-09

**Authors:** Ayoub Sabr, Ulrich Opoko, Mohamed Raiteb, Amina Maadane, Faiçal Slimani

**Affiliations:** a Faculty of Medicine and Pharmacy, Hassan II University of Casablanca, B.P, 5696, Casablanca, Morocco; b Oral and Maxillofacial Surgery Department, CHU Ibn Rochd, B.P, 698, Casablanca, Morocco

**Keywords:** Subcutaneous tuberculous cold abscess, Cervicofacial localization, Surgical drainage

## Abstract

**Introduction:**

Subcutaneous tuberculous cold abscesses represent a rare form of extra-pulmonary tuberculosis and their cervicofacial localization is exceptional. The management of this unusual form and location is medico-surgical and must be adapted to avoid progression to complications.

**Case report:**

We report the case of a double cervicofacial localization of subcutaneous tuberculous cold abscesses in an immunocompetent patient followed for pulmonary tuberculosis who benefited from a surgical drainage of the two abscesses with anti-tuberculosis treatment with good clinical evolution.

**Discussion:**

The diagnosis of subcutaneous tuberculous cold abscesses is based on a combination of anamnestic, clinical and paraclinical findings. Cold abscesses are most commonly described in patients with disseminated tuberculosis or during human immunodeficiency virus infection, but they may also occur in immunocompetent subjects. Monofocal localization is the most common and the association of several localizations is unusual. The treatment is medical-surgical, combining surgical drainage with anti-tuberculosis treatment.

**Conclusion:**

Subcutaneous tuberculous cold abscesses should be considered in the presence of any stubborn collection occurring in a context of tuberculosis infection. Early diagnosis is the best guarantee of a cure without complications.

## Highlights

• Subcutaneous tuberculous cold abscess is a rare presentation of extra-pulmonary tuberculosis.
• Cervico-facial localization is exceptional.
• The association of two locations is unusual.
• The diagnosis requires the comparison of a set of anamnestic, clinical and paraclinical arguments.
• The therapeutic management must be adapted to avoid the evolution towards complications.

## 1 Introduction

Tuberculosis is an infectious disease that is endemic in developing countries [1]. It can affect all organs of the body and can be localised to a single organ or disseminated with various clinical manifestations. Human-to-human transmission occurs via the airborne route from a person with microscopy-positive pulmonary tuberculosis (MPT+) to a healthy person. Only the pulmonary form of tuberculosis is contagious. Subcutaneous cold abscess tuberculosis is a rare form of tuberculosis that most often occurs in the context of severe disseminated tuberculosis or complicates pleuropulmonary or osteoarticular tuberculosis [2]. Cervicofacial localization of cold tuberculosis abscesses is rare and few cases have been published in the literature. They require specific and adapted management based on surgical drainage accompanied by anti-bacillary treatment in order to avoid any complications and improve the prognosis.

This work has been reported in line with the SCARE 2020 criteria [3].

## 2 Case report

A 33 year old female patient, known to have microscopy positive pulmonary tuberculosis (MPT+) complicated by a left pneumothorax, under anti-tuberculosis treatment having received two months of quadritherapy based on Rifampicin + Isoniazid + Pyrazinamide + Ethambutol and then during the second phase of treatment with dual therapy based on Rifampicin + Isoniazid (one month), referred to our department of maxillofacial surgery for two swellings, one fronto-glabellar and the other posterior right cervical, progressively evolving over the last 4 months, initially frontal and then secondarily right cervical, evolving in a context of asthenia, anorexia, evening fever and weight loss of 13 kg in 6 months.

**Fig. 1:**
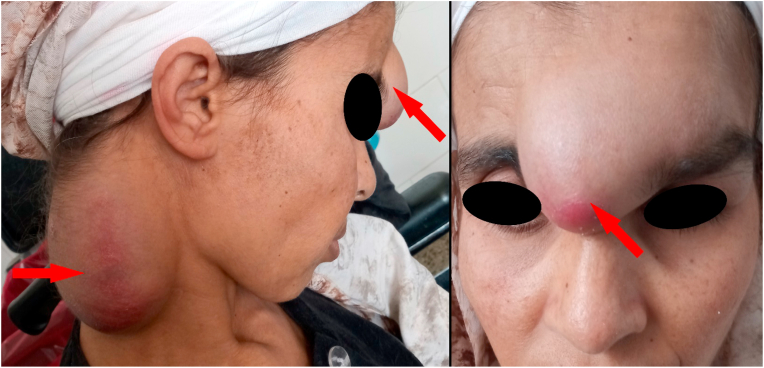
Clinical presentation of the fronto-glabellar and posterior right cervical swellings.

Clinical examination found two swellings of soft consistency, fluctuating on palpation, painless, warm and fairly well limited [Fig. 1]. Examination of the lymph nodes did not reveal any cervical or distant adenopathies and pleuropulmonary auscultation found bronchial rales on the right and an abolition of vesicular murmurs on the left as a consequence of her pulmonary tuberculosis.

**Fig. 2:**
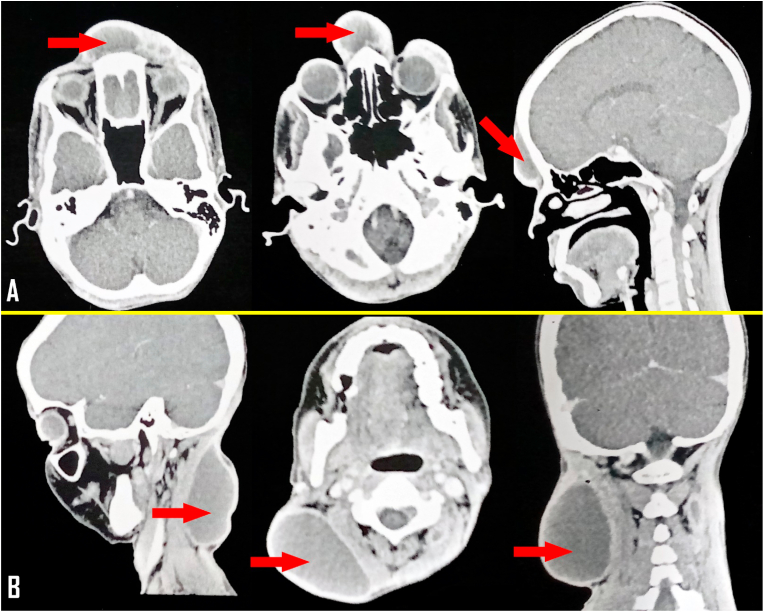
Cranio-cervico-facial CT scan showing a fronto-glabellar and right posteriour cervical subcutanous collections.

Cranio-cervico-facial CT scan showed two superficial subcutaneous collections in the fronto-glabellar and right posterior cervical regions, with no intracranial abnormalities suggestive of fronto-glabellar and cervical abscesses [Fig. 2].

**Fig. 3:**
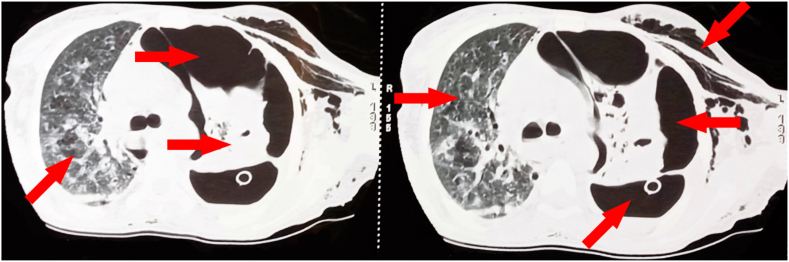
Chest CT scan showing a sequellae lung with left pneumothorax.

Chest CT scan revealed a sequelae lung, with parenchymal dystrophy with excavations and para septal emphysema on the right side and a medium-sized pneumothorax with underlying lung collapse on the left side [Fig. 3].

**Fig. 4:**
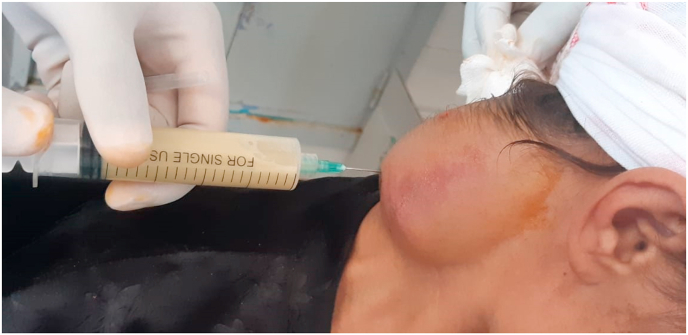
exploratory puncture showing the purulent content of the cervical swelling.

An exploratory puncture of the two fronto-glabellar and cervical swellings confirmed the diagnosis of abscess by bringing back a purulent fluid [Fig. 4]. Cytobacteriological examination of the samples confirmed the tuberculous origin of the two abscesses by the detection of Acid-Alcohol-Resistant Bacilli (AARB) on direct examination, followed by positivity of the Xpert gene by polymerase chain reaction (PCR) and isolation of Mycobacterium tuberculosis hominis by culture. The diagnosis of multifocal tuberculosis was retained in the patient, and then a human immunodeficiency virus serology was carried out on the patient, which came back negative, proving her immunocompetence.

After the results of the cyto-bacteriological analysis, the patient underwent surgical drainage of the two abscesses and was referred back to the pneumology department for further management and therapeutic readjustment. Anti-tuberculosis treatment was maintained in the patient for a total of 12 months. The follow-up at the consultation was done at D-14, 1 month, 2 months and 3 months by careful clinical examination alone without imaging, with a good evolution without signs of local or distant recurrence.

## 3 Discussion

Subcutaneous tuberculous cold abscesses are a rare and unusual form of tuberculosis accounting for 1% of the extra pulmonary form of the disease [4]. They are most commonly described in patients with severe and disseminated tuberculosis, especially during human immunodeficiency virus (HIV) infection [[5], [6], [7]]. Cervicofacial localization is exceptional and may be either isolated or associated with other forms of tuberculosis.

The pathogenesis of cold abscesses of tuberculosis origin is not univocal. Inoculation may be direct by transcutaneous route of the tuberculosis agent, especially in agricultural environments. Hematogenous dissemination in the blood or lymphatic system is evoked for forms complicating miliary tuberculosis or associated with other extra-pulmonary or multifocal bone diseases, contiguous extension of a cervical tuberculous adenitis is the most predominant mechanism of cervical subcutaneous tuberculous abscesses [2,5,6,8]. Three risk factors have been identified as predisposing to extrapulmonary tuberculosis: black race, female gender and immunosuppression [7].

Confirmation of the diagnosis of Subcutaneous tuberculous cold abscesses is based on a combination of anamnestic, clinical and paraclinical findings. A history of tuberculosis or concomitant active tuberculosis is often found. Their clinical presentation is aspecific. They are characterised by an insidious onset leading to a delay in diagnosis and are manifested by a soft swelling that rarely fluctuates, rarely suggesting an infectious origin, with fistulisation of the skin in the later forms. Tuberculous abscesses are often single, but may present with two or more sites [5,6,9]. Medical imaging is useful for diagnosis, although the radiological presentation of subcutaneous tuberculous cold abscesses is non-specific. Ultrasound can be used to determine the echogenicity of a swelling seen on clinical examination. CT scan and magnetic resonance imaging (MRI) can be used to study the locoregional extension of the abscess and to search for other locations that are inaccessible on clinical examination [5,6,9]. In our case, the patient was known to have MPT + pulmonary tuberculosis in the second phase of her anti-tuberculosis treatment, the liquid nature of the two fronto-glabellar and cervical swellings was confirmed by cranio-cervicofacial CT scan which eliminated the presence of other deep anomalies.

The diagnosis of tuberculosis in abscesses is based on the isolation of the mycobacterium in the puncture fluid and/or in the biopsy fragments or on the histological and bacteriological study of the surgical excision specimens with culture. Bacteriological examination allows the diagnosis to be confirmed by showing Acid-Alcohol-Resistant Bacilli (AARB) on direct examination and Mycobacterium tuberculosis on culture. Evidence of the germ on direct examination of the pus is rare, which explains the delay in treatment, hence the interest of the polymerase chain reaction (PCR) using the Xpert gene. The latter allows a rapid diagnosis and therefore the initiation of a standard anti-tuberculosis treatment that can be modified later according to the results of the cultures [5,6,10]. In our case, the diagnosis of abscess was made by puncturing the two collections, which yielded a purulent fluid. The tuberculosis origin of the abscesses was then confirmed by the detection of AARB on direct examination, followed by the positivity of the Xpert gene on PCR and the isolation of Mycobacterium tuberculosis hominis on culture. In our case, the diagnosis of multifocal tuberculosis in an immunocompetent subject was retained. Several hypotheses have been put forward to explain the occurrence of this diffuse form of tuberculosis in immunocompetent individuals. Some authors have been able to establish a relationship between diffuse tuberculosis and the intensity of transmission in the community. Others have implicated malnutrition as a contributing factor. Cathérinot described the Mendelian susceptibility syndrome to mycobacterial infections by the existence of defects in the interleukin-12-interferon-gamma axis and exposing to diffuse tuberculosis [11,12].

The therapeutic management of cold abscesses of tuberculous origin combines surgery and anti-tuberculosis therapy. The surgical procedure is both diagnostic and therapeutic, allowing the abscess to be drained in its entirety and the underlying necrotic tissue to be removed, with biopsies being taken to obtain histological evidence. The association of anti-tuberculosis treatment with surgery is recommended in order to reduce local recurrence. Anti-tuberculosis treatment protocol includes an initial phase of two months of quadruple therapy: Rifampicin (10mg/kg/d) + Isoniazid (5mg/kg/d) + Pyrazinamide (20–30 mg/kg/d) + Ethambutol (15–20 mg/kg/d), followed by dual therapy: Rifampicin + Isoniazid for a total duration of treatment of 9–12 months [[2], [5], [6], [9]]. In our case, surgical drainage of both abscesses was performed immediately after confirmation of the diagnosis with maintenance of anti-tuberculosis treatment for a total period of 12 months.

The prognosis depends on the delay in diagnosis and the rapidity of the initiation of treatment. It is usually favourable. Complete resection of the tuberculous cold abscess with excision of the underlying necrotic tissue reduces the rate of recurrence. This surgery, coupled with anti-tuberculosis treatment, is the only guarantee of a definitive cure [[4], [5], [6]].

## 4 Conclusion

Subcutaneous tuberculous cold abscesses are a rare form of extra pulmonary tuberculosis whose cervicofacial localization is exceptional. Its confirmation requires a combination of anamnestic, clinical and paraclinical elements. Early diagnosis and appropriate management are the only means of avoiding complications.

## Provenance and peer review

Not commissioned, externally peer reviewed.

## Ethical approval

Written informed consent was obtained from the patient for publication of this case report and accompanying images. A copy of the written consent is available for review by the Editor-in-Chief of this journal on request.

## Sources of funding

The authors declared that this study has received no financial support.

## Consent

Written informed consent was obtained from the patient for publication of this case report and accompanying images. A copy of the written consent is available for review by the Editor-in-Chief of this journal on request.

## Registration of research studies

Name of the registry:
Unique Identifying number or registration ID:
Hyperlink to your specific registration (must be publicly accessible and will be checked):

## Guarantor

Ayoub Sabr

Ayoub Sabr: Corresponding author writing the paper

Ulrich Opoko: writing the pape

Mohamed Raiteb: writing the paper

Amina Maadane: writing the paper

Faiçal Slimani: Correction of the paper

## Declaration of competing interest

Authors of this article have no conflict or competing interests. All of the authors approved the final version of the manuscript.
